# Seasonal, gender and regional variations in total phenolic, flavonoid, and condensed tannins contents and in antioxidant properties from *Pistacia atlantica* ssp. leaves

**DOI:** 10.1080/13880209.2017.1291690

**Published:** 2017-02-28

**Authors:** Ziyad Ben Ahmed, Mohamed Yousfi, Johan Viaene, Bieke Dejaegher, Kristiaan Demeyer, Debby Mangelings, Yvan Vander Heyden

**Affiliations:** a Laboratory of Fundamental Science, University Amar Telidji, Laghouat, Algeria;; b Department of Analytical Chemistry and Pharmaceutical Technology, Vrije Universiteit Brussel (VUB), Brussels, Belgium;; c Laboratory of Instrumental Analysis and Bioelectrochemistry, Université Libre de Bruxelles (ULB), Bruxelles, Belgium;; d Department of Toxicology, Dermato-Cosmetology and Pharmacognosy, Vrije Universiteit Brussel (VUB), Brussels, Belgium

**Keywords:** Total phenolic content, flavonoids content, condensed tannins content, DPPH assay, RPC assay, *Pistacia atlantica* leaves

## Abstract

**Context:** The widespread use of *Pistacia atlantica* Desf. ssp. (Anacardiaceae) in traditional medicine can be partly attributed to the content of its secondary metabolites, in particular, the phenolic compounds.

**Objective:** The effects of harvest period, growing region and gender on the phenolic compounds, flavonoids and condensed tannins contents were studied, as well as on the antioxidant activities of *P. atlantica* leaves in order to provide a scientific basis for optimal collection.

**Materials and methods:** Leaves were collected monthly from April to October 2010 in two Algerian sites. The powdered leaves were used for preparing the ethyl acetate extract. Contents of total phenolics (TPC), flavonoids (FC) and condensed tannins (CTC) were determined spectrophotometrically. Antioxidant activity was evaluated through radical scavenging activity (RSA) of 2,2-diphenyl-1-picrylhydrazyl (250 μM) and the reducing power capacity (RPC) determination by K_3_Fe(CN)_6_ (1%).

**Results:** The TPC was found to vary from 79 ± 13 to 259 ± 8 mg gallic acid equivalents/g of dry weight (DW) during the study period. The RSA and RPC varied between 262 ± 18 and 675 ± 21 mg Ascorbic Acid Equivalent (AAE)/g DW, and from 259 ± 16 to 983 ± 20 mg AAE/g DW, respectively. A seasonal pattern was observed consisting of a decrease in TPC content and RPC from spring to autumn. The FC, CTC and RSA did not show a seasonal pattern.

**Discussion and conclusion:** Our findings showed that secondary metabolite content and antioxidant activities of *P. atlantica* leaves were more influenced by harvest time and growing region than by gender.

## Introduction

The use of synthetic antioxidants is currently under reconsideration because of their potential toxicological risks (Nieva‐Echevarría et al. 2015). Accordingly, researchers are seeking for natural antioxidants originating from plants, which have a greater benefit than the synthetic (Adhami & Muktar 2013). Natural antioxidants are substances capable of preventing or delaying the oxidative stress even at relatively small concentrations by acting directly on reactive oxygen species or by stimulating endogenous defense systems. When there is an imbalance between the oxidation and antioxidation processes, an excess of free radicals may accumulate that can cause oxidative damage to biological molecules and tissues resulting in many diseases in humans (Liu & Huang 2014).

Many bioactive principles from plants have been reported to exhibit strong antioxidant activities. Phenolic compounds constitute one of the most numerous and ubiquitous groups, of the secondary metabolites. They defend the plants against a variety of herbivores or aggression by pathogens (Brunetti et al. 2015). Researchers explored that these antioxidants may play an important role in the prevention of free-radical-induced diseases by acting as hydrogen and/or electron donors, which quench the free radicals and are then converted to antioxidant radicals (Nyanhongo et al. 2013). Epidemiological studies have revealed that phenolic compounds provide a significant protection against the development of several chronic diseases, such as cardiovascular diseases, cancer, diabetes, infections, aging and asthma (Liu & Huang 2014).

The genus *Pistacia* (Anacardiaceae) consists of at least 11 species. Plants of this genus are primarily distributed in the Northern hemisphere and include both evergreen and deciduous species *Pistacia. atlantica* Desf. ssp. is one of the most widely distributed wild species, called ‘Butom’ in Algeria, and is the most characteristic plant species of the arid and semi-arid regions of the country (Belhadj 2002; El Zerey-Belaskri & Benhassaini 2016).

*Pistacia atlantica* is believed to have several therapeutic properties, such as relieving upper abdominal discomfort and pain, dyspepsia and peptic ulcer (Zarshenas et al. 2013). Scientific findings also revealed the wide pharmacological activities such as antioxidant, antimicrobial and antihyperglycemic activities from various parts of this species (Benhammou et al. 2008; Ben Ahmed et al. 2016). The widespread use of *P. atlantica* in traditional medicine can be partly attributed to the content of the phenolic compounds.

The phenolic profiles of plant tissues are known to be affected by many factors, such as genotype, environment, growth stage, time of harvesting, process and storage conditions, and method of analysis (Vagiri et al. 2015). Although the phenolic composition of *P. atlantica* leaves is reported (Ben Ahmed et al. 2016), there is little or no knowledge among local cultivators for choosing the optimal harvest time associated, for instance, with a maximal potency in antioxidant capacity.

Hence, the aim of this study was to evaluate the fluctuations in total phenolic (TPC), flavonoid (FC) and condensed tannins content (CTC) in extracts of *P. atlantica* leaves. Antioxidant activity was evaluated by both the radical scavenging DPPH (2,2-diphenyl-1-picrylhydrazyl) and the reducing power capacity (potassium ferricyanide) methods. The relationships between antioxidant activities and contents of TPC, FC and CTC, measured in the plant samples, are discussed in order to provide a scientific basis for an optimal usage of *P. atlantica* leaves.

## Materials and methods

### Chemicals

Ethyl acetate, petroleum ether (boiling point 40–60 °C), HCl (37%), FeCl_2_·4H_2_O, FeCl_3_·6H_2_O, butanol, methanol, and 2,2-diphenyl-1-picrylhydrazyl (DPPH^•^) were obtained from Sigma-Aldrich (Munich, Germany). Folin–Ciocalteau reagent, vanillin and hydrogen peroxide solution (30% w/w) were purchased from Sigma (Steinheim, Germany), and potassium phosphate (monobasic and dibasic), sodium carbonate and quercetin from Fluka (Buchi, Switzerland). Trichloroacetic acid (TCA), gallic acid and catechin were obtained from Riedel-de Haen (Seelze, Germany), and ascorbic acid from Panreac (Barcelona, Spain).

### Plant materials

The identity of the leaf samples was confirmed by Prof. Safia Belhadj (Department of Agropastoralism, Faculty of Science, Achour Zian University, Djelfa, Algeria), and voucher specimen (LM: Laghouat male leaves, LF: Laghouat female leaves, OM: Ain oussera male leaves, and OF: Ain oussera female leaves) are deposited at the Department of Biology, University of Laghouat (Algeria).

Both female and male trees were sampled each from the lower, central and upper parts in about equal amounts. Leaves of one gender (collected from five randomly selected trees) were carefully mixed, dried and then smashed manually, before storing them in polyethylene bags at ambient temperature in the dark until use. The leaves were smashed because higher extraction yields of phenolics are achieved when extracting from smaller particles (Fonseca et al., 2007). The dried leaves of a green color, not red, were used. The leaves were collected monthly from April until October (2010), at the middle of each month. Trees with a similar age were sampled from two growing regions chosen along a transect of increasing aridity: Ain oussera (medium arid) and Laghouat (arid) located at 200 and 400 km, respectively, south of Algiers, Algeria. Plants density and height of the trees were similar for the two sites. The two locations have same the kind of soil, which is clay. This soil is composed of tiny particles that are hard and able to become easily impacted. The clay soil locally known as ‘Daya’. About 20 g of leaves were harvested from each individual tree.

### Information on weather conditions

Data recorded during 2010 (April–October) at the station L'Mekrareg Airport (33.76°N, 2.93°E, altitude 765 m) and KsarChellal Airport (35.17°N, 2.32°E, altitude 801 m) for Laghouat and Ain oussera regions, respectively, were provided by the Weather Underground (http://french.wunderground.com/history/) (Table 1).

**Table 1.** Average monthly temperature (T), day length (DL) and visible light length (LVL) in Laghouat and Ain-oussera during 2010.

Variable	Region	April	May	June	July	August	September	October
T (°C)	Laghouat	19	20	28	32	31	25	19
	Ain oussera	13	15	21	27	26	21	16
DL (h)	Laghouat	13.01	13.54	14.23	14.11	13.25	12.23	11.21
	Ain oussera	13.03	13.58	14.28	14.15	13.28	12.24	11.20
LVL (h)	Laghouat	13.52	14.49	15.21	15.07	14.17	13.13	12.11
	Ain oussera	13.55	14.54	15.27	15.13	14.21	13.14	12.10

### Sample preparation

The method described by Boeing et al. (2014) and Yousfi et al. (2006) was employed to prepare the crude extract of *P. atlantica* leaves. Each sample (2 g) was macerated in 100 mL acetone/water (v/v, 7:3) in the dark for 72 h, followed by filtration through a Whatman filter paper (Grade 1: 11 μm, Sigma**-**Aldrich, France). The acetone was removed by using a rotary evaporator (temperature 50 °C). The aqueous phase was subsequently extracted with 100 mL petroleum ether. The lower (aqueous phase) was recovered and the extraction repeated three times for the removal of pigments and most of the lipids (dissolved into the upper phase). The lower aqueous phase was then extracted twice with 100 mL ethyl acetate, in the presence of 1 mL ammonium sulfate solution (20%, w/v) and 1 mL *meta*-phosphoric acid (2%, v/v). The ethyl acetate fraction was dried by adding 2 g anhydrous sodium sulfate, filtered and evaporated to dryness using a rotary evaporator (temperature 50 °C). The residue was redissolved in 10.0 mL absolute methanol and stored at −5 °C until analysis. In the following assays absorbances were measured on a Shimadzu UV 160A spectrophotometer (Kyoto, Japan) and each sample was prepared in triplicate and the mean value calculated.

### Determination of total phenolic contents (TPC) Folin–Ciocalteu method (FCM)

The total phenolic content was determined with the Folin–Ciocalteu reagent based on the method described in Lou et al. (2014). A calibration curve was obtained using gallic acid as standard. Different concentrations of gallic acid (0.05–0.35 mg/mL) were prepared in methanol/water (60:40, v/v). A sample 5 fold**-**diluted in methanol (100 μL) was added to a test tube. Both standards and samples were mixed with 500 μL 10-fold-diluted Folin–Ciocalteu reagent in water and 2 mL aqueous sodium carbonate solution (4%, w/v). The final mixture was shaken and then incubated for 30 min in the dark at room temperature. The absorbance of all standards and samples was measured at 760 nm, and the results expressed as milligrams of gallic acid equivalents (GAE) per gram of dry leaf weight.

### Determination of flavonoid content (FC)

The FC in the extracts was determined spectrophotometrically according to the method described by Vongsak et al. (2013). It is based on the formation of a flavonoid aluminum complex, showing an absorbance maximum at 430 nm. Quercetin was used to set up the calibration curve in methanol (0.005–0.025 mg/mL). Standard and sample from 10 fold-diluted extract in methanol (1 mL) were mixed with 1.0 mL of methanolic aluminum chloride solution (2%, w/v). After incubation at room temperature for 15 min, the absorbance was measured and the flavonoid content was expressed in mg quercetin equivalents (QE) per g dry leaf weight.

### Determination of condensed tannins content (CTC)

Evaluation of total CTC was carried out as described by Chupin et al. (2013). The extracts (1 mL) were placed in two separate test tubes (one for the sample and one for the blank). Methanol vanillin solution (4%, w/v) (3 mL) and 1.5 mL concentrated hydrochloric acid were added to the sample tube, while 3 mL pure methanol and 1.5 mL concentrated hydrochloric acid were added to the blank tube. The mixtures were kept for 15 min in the dark at room temperature; then the absorbance was measured at 500 nm. A standard curve was prepared with (+)-catechin (0.5–3.5 mg/mL). The results were expressed as milligrams of (+)-catechin per gram of dry leaf weight.

### Evaluation of antioxidant activity

#### DPPH (2,2-diphenyl-1-picrylhydrazyl) assay

The DPPH assay was performed according to Du et al. (2014). The methanolic DPPH^•^ solution (250 μM) was prepared daily. Then, 1 mL of 25-, 50-, or 100-fold diluted extract in methanol was mixed with 1 mL of DPPH solution and incubated for 30 min in the dark. Immediately after mixing at time *t* = 0 min (t_0_) and after 30 min, the absorbances were measured at 517 nm. A standard curve was prepared by determining the decrease in absorbance of the DPPH radical solution over a period of 30 min with different concentrations of ascorbic acid (0.002–0.016 mg/mL). The DPPH radical scavenging activity (RSA) was defined as ascorbic acid equivalent antioxidant capacity (AAEAC) and expressed as mg ascorbic acid equivalents per gram of dry leaf weight (Leong & Shui 2002). AAEAC was calculated using the following equation:

$$\mathrm{AAEAC}=\left(\Delta A_{\mathrm{sample}}/K\right)\times \;D\;\times \;V\;\times \left(1/W_{\mathrm{sample}}\right)$$

where Δ*A*_sample_ is the change in absorbance in the presence of extract, *K* the slope of the standard curve, *D* the dilution factor (25, 50 or 100) of the extract, *V* the total extract volume (10 mL) and *W*_sample_ the sample weight used for extraction (g).

#### Potassium ferricyanide [K_3_Fe(CN)_6_] reducing power capacity (RPC)

The reducing power of the *P. atlantica* leaf extracts was determined according to the method described by Kannan et al. (2013). 50-, 100-, and 200-fold methanol-diluted extracts (2.5 mL) were mixed with 2.5 mL phosphate buffer 200 mM at pH 6.6 and 2.5 mL of 1% potassium ferricyanide. The mixture was incubated at 50 °C for 20 min. Then, 2.5 mL of TCA (10% w/v) was added and the mixture was centrifugated for 10 min at 1000 *g* (Universal 32 R, Hettich, Tuttlingen, Germany). The supernatant (2.5 mL) was added to glass tubes containing 2.5 mL distilled water and 0.5 mL FeCl_3_·6H_2_O (0.1%, w/v). The absorbance of the resulting solution was measured at 700 nm using distilled water as control solution. The reducing power activity was calculated from a standard curve based on a set of ascorbic acid solutions (0.05–0.3 mg/mL). Results were defined as ascorbic acid equivalent antioxidant capacity (AAEAC) and expressed as milligrams of ascorbic acid equivalents per gram of dry leaf weight.

### Statistical analysis

One-way analyses of variance (ANOVA) followed by a Student–Newman Keuls (SNK) *post hoc* test were used to estimate the significance of the factor harvest month, gender and growing region on the levels of TPC, FC, CTC and AAEAC. Paired *t*-tests were used to compare the differences in the averages of TPC, FC, CTC and AAEAC between harvest months, gender and growing region. Calculations were performed using SPSS (version 16, SPSS, Prentice Hall, Chicago IL, 2007). Principal Component Analysis (PCA) was performed, using XLSTAT-Pro version 2014 (Addinsoft, New York, NY).

## Results

**Figure 1.**
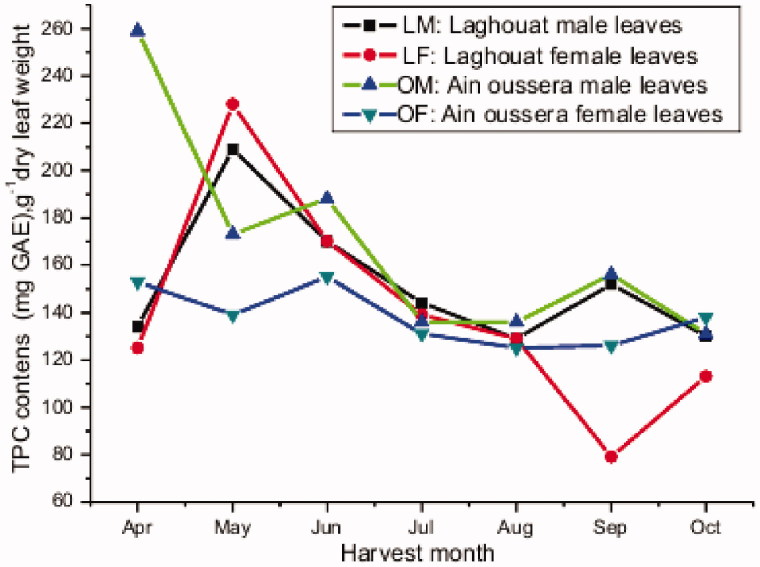
Monthly variation in the total phenolic contents.

In order to evaluate seasonal changes in the total phenolic content, a systematic analysis was carried out by sampling leaves as described in ‘Materials and methods’ section. The TPC varied over the studied period as shown in Figure 1 and Table 2. A maximum seems to be reached in spring. In Ain oussera the maximum is observed earlier (April) than in Laghouat (May), regardless of the fact that temperatures in Laghouat are systematically higher. Then over summer towards autumn, the TPC seems to decrease.

**Table 2.** Total phenolic contents in *P. atlantica* leaves harvested in different months from Laghouat and Ain oussera regions, expressed as mg gallic acid quivalents (mg GAE)/g dry leaf weight.

Season	Month	LM ± SD (*n* = 3)	LF ± SD (*n* = 3)	OM ± SD (*n* = 3)	OF ± SD (*n* = 3)
Spring	April	136^a^ ± 4	124^b^ ± 10	258^d^ ± 8	151 ± 8
	May	208^c^ ± 8	228^d^ ± 21	172^bc^ ± 15	144 ± 13
Summer	June	173^b^ ± 13	169^c^ ± 13	188^c^ ± 6	154 ± 15
	July	144^a^ ± 10	138^bc^ ± 17	135^a^ ±13	131 ± 15
	August	129^a^ ± 16	128^b^ ± 19	135^a^ ± 8	125 ± 23
Autumn	September	152^a^ ± 10	79^a^ ± 13	155^ab^ ± 24	125 ± 7
	October	130^a^ ± 5	112^b^ ± 30	130^a^ ± 10	137 ± 17

Values in one column followed by the same superscript do not differ significantly according to SNK *post hoc* test. Superscripts a→ b→ c: indicate increasing concentrations.

Possible influences of the factors under study (gender, harvest time, and growing region) were evaluated. ANOVA was performed for each region and gender, to evaluate the effect of the harvest month on the TPC. In both regions, a significant difference was found in the TPC levels between different harvest months within each gender (*p* < 0.0001), with the exception of the female leaves of the Ain oussera region (*p* = 0.151). Furthermore, paired *t*-tests were performed on the monthly averages to evaluate systematic differences between both genders, collected within each region, and between regions.

Gender had no significant effect on TPC of *P. atlantica* leaves (*p* = 0.164 for Laghouat and *p* = 0.082 for Ain oussera region) over the considered period. Thus both genders of *P. atlantica* leaves growing within each region had a similar behavior according to variation of TPC. However, the results for the Ain oussera region are rather borderline non-significant. From Figure 1, one would conclude that in spring (April) the male leaves seem to have a higher amount, but later the differences decrease and disappear.

To evaluate the differences in TPC concentration between Laghouat and Ain oussera regions, the second paired *t*-test was applied. The fluctuations in TPC were found to be similar for both growing regions (*p* = 0.807). Thus over the considered period the overall TPC profile is rather similar.

**Figure 2.**
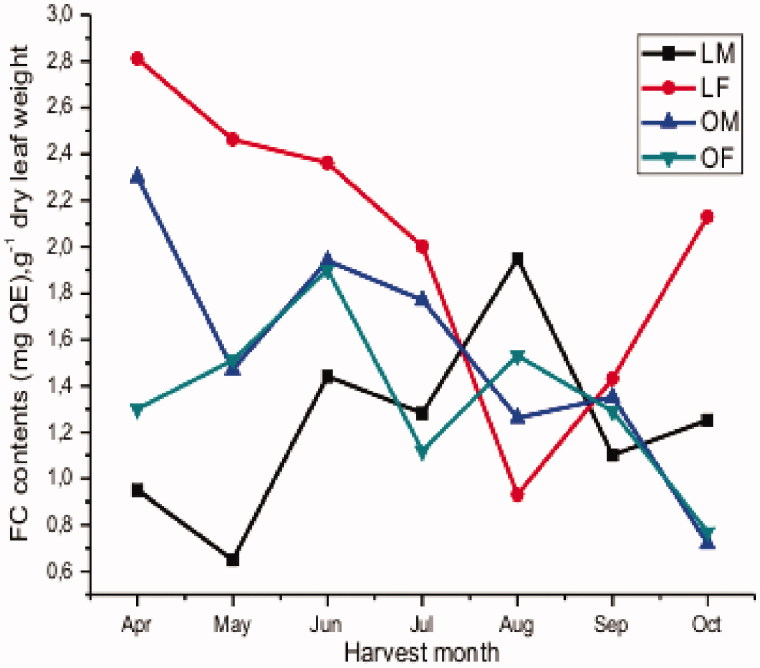
Monthly variation in the flavonoid content.

Flavonoids are widely distributed plant secondary metabolites (Stanković et al. 2015). They have generated interest because of their broad human health promoting effects, most of which are related to their antioxidant properties and to synergistic effects with other antioxidants (Li et al. 2015). Figure 2 and Table 3 show the monthly variation in total flavonoid content measured in the leaves of *P. atlantica*. A less pronounced tendency than for the TPC is seen.

**Table 3.** Flavonoid content in *P. atlantica* leaves, harvested in different months from Laghouat and Ain oussera regions, expressed as mg quercetin equivalents (mg QE)/g dry leaf weight.

Season	Month	LM ± SD (*n* = 3)	LF ± SD (*n* = 3)	OM ± SD (*n* = 3)	OF ± SD (*n* = 3)
Spring	April	0.95^ab^ ± 0.06	2.81^c^ ± 0.88	2.30^c^ ± 0.16	1.30^bc^±0.13
	May	0.65^a^ ± 0.10	2.46^bc^ ± 0.24	1.47^b^ ± 0.10	1.51^c^ ± 0.14
Summer	June	1.44^b^ ± 0.29	2.36^bc^ ± 0.22	1.94^bc^ ± 0.71	1.90^d^ ± 0.11
	July	1.28^ab^ ± 0.06	2.00^bc^ ± 0.23	1.77^bc^ ± 0.09	1.12^b^ ± 0.14
	August	1.95^c^ ± 0.50	0.93^a^ ± 0.12	1.26^ab^ ± 0.13	1.53^c^ ± 0.25
Autumn	September	1.10^ab^ ± 0.30	1.43^ab^ ± 0.43	1.35^ab^ ± 0.20	1.29^bc^ ± 0.18
	October	1.25^ab^ ± 0.25	2.13^bc^ ± 0.26	0.72^a^ ± 0.17	0.73 ^a^± 0.03

Values in one column followed by the same superscript do not differ significantly according to SNK *post hoc* test. Superscripts a→ b→ c: indicate increasing concentrations.

The potential influences of the main factors region, gender and harvest month were examined as for the total phenolic content. The ANOVA showed a significant effect of harvest months on FC for each region and gender (*p* < 0.0015). The paired *t*-test for monthly averages showed no significant systematic difference in FC between male and female leaves, collected within each region (*p* = 0.095 for Laghouat and *p* = 0.275 Ain oussera). The factor gender did not have a significant effect on the level of FC in *P. atlantica* leaves over the considered period, even though in the Laghouat region the FC concentration in spring and early summer seems higher in the male leaves.

The paired *t*-test on the monthly regional averages, showed no statistically significant difference in FC (*p* = 0.446). It can therefore be concluded that FC seems to behave similarly in both regions.

**Figure 3.**
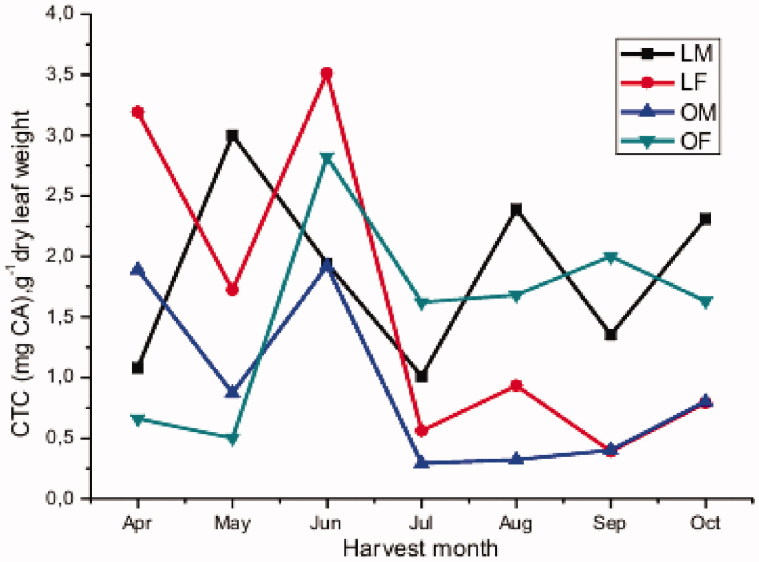
Monthly variation in condensed tannins content.

Condensed tannins (proanthocyanidins) comprise a group of polyhydroxyflavan-3-ol oligomers and polymers linked by carbon–carbon bonds between flavanol subunits (da Silva et al. 2014). The most common classes are the procyanidins, which are chains of catechin, epicatechin and their gallic acid esters, and the prodelphinidins, which consist of gallocatechin, epigallocatechin and their galloylated derivatives as monomeric units (Suriano et al. 2015). Condensed tannins have attracted great attention due to the rapid growing evidence associating these compounds with a wide range of potential health benefits (de-Faria et al. 2012). The results of the estimation of the condensed tannins contents in the leaves of *P. atlantica* are depicted in Figure 3 and Table 4. Variations in the average CTC were observed in both genders and both regions over the harvesting period. However, no real tendency is observed. The ANOVA revealed significant differences in CTC (*p* ≤ 0.0001) between different harvest months for each region and gender. The paired *t-*test for monthly averages reported no statistically significant difference in CTC between both genders collected in a given each region (*p* = 0.983 for Laghouat and *p* = 0.161 for Ain oussera). Overall, findings suggest that leaves of *P. atlantica* do not systematically differ in CTC between genders.

**Table 4.** Condensed tannin contents in *P. atlantica* leaves, harvested in different months from Laghouat and Ain oussera regions, expressed as mg catechin equivalents (mg CAE)/g dry leaf weight.

Season	Month	LM ± SD (*n* = 3)	LF ± SD (*n* = 3)	OM ± SD (*n* = 3)	OF ± SD (*n* = 3)
Spring	April	1.10^a^ ± 0.22	3.19^c^ ± 0.94	1.89^a^ ± 0.14	0.66^a^ ± 0.06
	May	3.00^c^ ± 0.23	1.72^b^ ± 0.39	0.87^b^ ± 0.09	0.50^a^ ± 0.05
Summer	June	1.94^b^ ± 0.50	3.51^c^ ± 0.48	1.92^c^ ± 0.49	2.82^c^ ± 0.68
	July	1.01^a^ ± 0.37	0.56^a^ ± 0.13	0.29^a^ ± 0.10	1.62^b^ ± 0.12
	August	2.39^b^ ± 0.17	0.93^ba^ ± 0.38	0.32^a^ ± 0.04	1.68^b^ ± 0.18
Autumn	September	1.35^a^ ± 0.16	0.39^a^ ± 0.03	0.40^a^ ± 0.04	2.00^b^ ± 0.47
	October	2.31^b^ ± 0.18	0.79^ba^ ± 0.09	0.80^b^ ± 0.05	1.63^b^ ± 0.16

Values in one column followed by the same superscript do not differ significantly according to SNK *post hoc* test. Superscripts a→ b→ c: indicate increasing concentrations.

The paired *t-*test showed no significant systematic difference in CTC between Laghouat and Ain oussera regions (*p* = 0.193). However, even though not significant, the results of Figure 3 and Table 4 indicate a tendency that the contents in the Laghouat region tended to be higher than these from Ain oussera.

**Figure 4.**
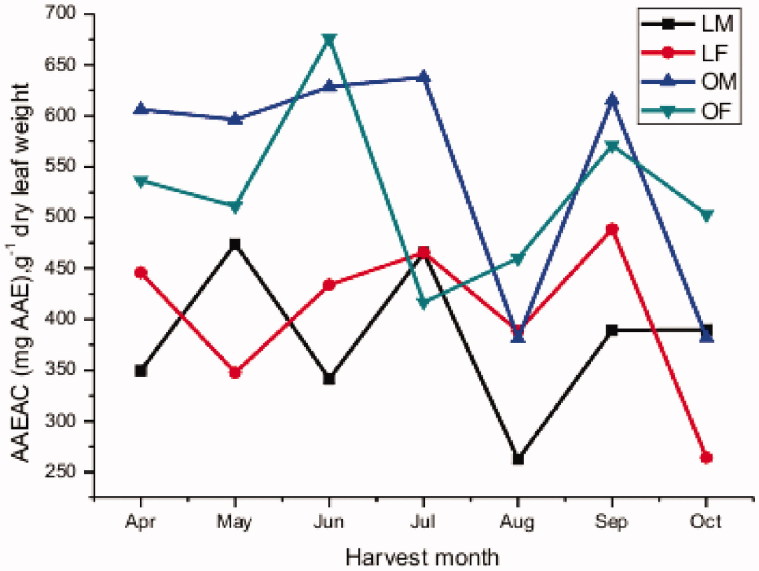
Monthly variation in the DPPH scavenging activity.

Recognition of many health benefits provided by phenolic compounds has encouraged increased scientific interest in determining the antioxidant capacity of various plant-derived products. However, a standardized method for the determination of the antioxidant properties has not yet been established. The total antioxidant capacity demands at least two methods to reflect and differentiate both the single electron transfer (SET) and the hydrogen atom transfer (HAT) phenomena (Alov et al. 2015). The present study used two methods to estimate the antioxidant capacity, i.e. DPPH (HAT) and K_3_Fe(CN)_6_ (SET) assays, which are commonly used in biological samples, applying different procedures. To our knowledge, there is no study on the effects of harvest months, growing region and gender on the total antioxidant activities of *P. atlantica* leaves. Figure 4 and Table 5 present the results of the monthly variation in RSA (from DPPH test) in the vegetative leaves of *P. atlantica*. Leaves from Ain oussera tend to be more active than those from the Laghouat region across the entire studied period.

**Table 5.** The monthly variation in the DPPH scavenging activity in *P. atlantica* leaves which were collected in Laghouat and Ain oussera regions, expressed as mg ascorbic acid equivalent (mg AAE)/g dry leaf weight.

Season	Month	LM ± SD (*n* = 3)	LF ± SD (*n* = 3)	OM ± SD (*n* = 3)	OF ± SD (*n* = 3)
Spring	April	349^bc^ ± 14	445^ef^ ± 20	605^b^ ± 17	536^cd^ ± 27
	May	474^d^ ± 19	347^b^ ± 12	596^b^ ± 12	510^bc^ ± 15
Summer	June	341^b^ ± 15	433^d^ ± 17	628^b^ ± 48	675^e^ ± 21
	July	465^d^ ± 26	465^ef^ ± 34	637^b^ ± 35	416^a^ ± 39
	August	262^a^ ±18	388^c^ ± 20	380^a^ ± 55	459^b^ ± 12
Autumn	September	388^c^ ±12	488^e^ ± 8	615^b^ ± 31	571^d^ ± 22
	October	389^c^ ± 25	263^a^ ± 30	381^a^ ± 47	502^bc^ ± 25

Values in one column followed by the same superscript do not differ significantly according to SNK *post-hoc* test. Superscripts a→ b→ c → d→ e → f: indicate increasing activities.

The ANOVA confirmed that these activities were significantly different and this in the four leaf types considered (*p* ≤ 0.0001). A paired *t*-test for monthly averages revealed that the RSA did not significantly systematically differ between both genders (*p* = 0.597 for Laghouat and *p* = 0.598 for Ain oussera).

Furthermore the paired *t-*test indicated statistically significant systematic difference in the RSA between Laghouat and Ain oussera (*p* < 0.0005), suggesting that the growing region affects RSA of *P. atlantica* leaves.

**Figure 5.**
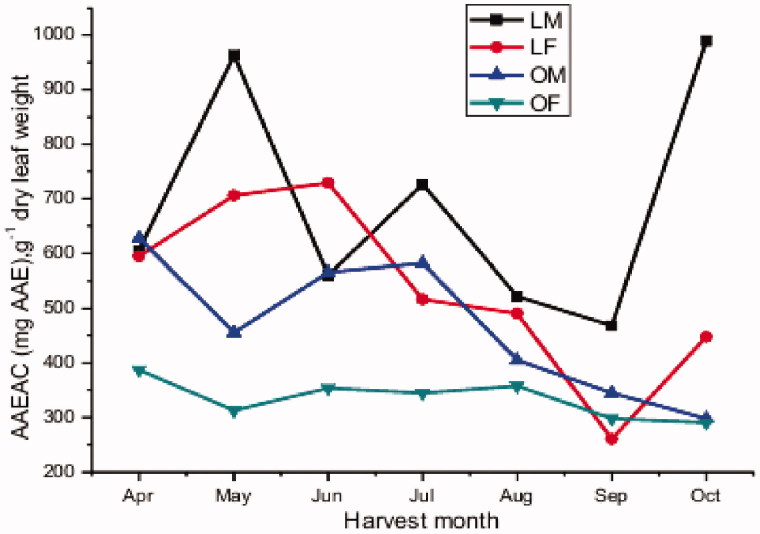
Monthly variation in the reducing power capacity.

For the RPC assay, i.e. a ferric ion-based assay, the presence of reducers (i.e. antioxidants) causes the reduction of the ferricyanide complex (Fe^3+^) to the ferrous form (Fe^2+^) by donating an electron. Monthly variations of the reducing power activity in *P. atlantica* leaves are shown in Figure 5 and Table 6. A variation in the reducing power activity was observed between regions, and occasionally gender and harvest time. A decreasing trend seems to occur over the measured period. The male leaves of Laghouat seem to have higher reducing power activity than the other of samples classes.

**Table 6.** The monthly variation in reducing power activity in *P. atlantica* leaves collected in Laghouat and Ain oussera regions, expressed as mg ascorbic acid equivalent (mg AAE)/g dry leaf weight.

Season	Month	LM ± SD (*n* = 3)	LF ± SD (*n* = 3)	OM ± SD (*n* = 3)	OF ± SD (*n* = 3)
Spring	April	604^c^ ± 20	594^d^ ± 27	627^f^ ± 16	386^a^ ± 21
	May	962^e^ ± 12	705^e^ ± 30	455^d^ ± 28	311^b^ ± 13
Summer	Jun	559^b c^ ± 33	728^e^ ± 31	564^e^ ± 21	353^b^ ± 19
	July	726^d^ ± 38	515^c^ ± 31	581^e^ ± 18	344^a^ ± 20
	August	520^ab^ ± 11	490^bc^ ± 26	404^c^ ± 16	356^a^ ± 12
Autumn	September	467^a^ ± 69	259^a^ ± 16	343^b^ ± 19	297^b^ ± 18
	October	989^e^ ± 20	447^b^ ± 45	297^a^ ± 23	289^b^ ± 22

Values in one column followed by the same superscript do not differ significantly according to SNK *post-hoc* test. Superscripts a→ b→ c → d→ e → f: indicate increasing activities..

ANOVA, revealed significant differences in reducing power between different harvest months and this for all genders and regions (*p* < 0.001). The paired *t*-test reported statistically significant systematic differences in AAEAC between both genders in the Ain oussera region (*p* = 0.012). This difference was not statistically significant in the Laghouat region (*p* = 0.119), even though the absolute differences often were much larger.

The paired *t*-test performed to evaluate activity differences between Laghouat region and Ain oussera, showed a significant difference (*p* = 0.002). It can therefore be concluded that the reducing power seems to behave differently in both regions.

In an attempt to identify potential compounds that contribute to the antioxidant activity of the extracts, Pearson correlation coefficients were calculated between the relative contents, including the TPC, FC and CTC, and the antioxidiant activities of RSA and RPC. The highest correlation was found between CTC and RPC (*r* = 0.483, *p* < 0.01), followed by that between TPC and RPC (*r* = 0.447, *p* < 0.01). However, neither significant correlations between FC on the one hand and RSA (*r* = 0.100, *p* > 0.05) or RPC (*r* = 0.180, *p* > 0.05) on the other, nor between CTC and RSA (*r* = −0.010, *p* > 0.05) were seen. In addition, our findings did not show any significant correlation between RSA and RPC results (*r* = −0.170, *p* > 0.05). Further, a better correlation was found between TPC/FC/CTC and the RPC assay, than with the RSA assay results.

## Discussion

The TPC in *P. atlantica* leaves were neither affected by the growing region nor the gender. The seasonal trend showed that the phenolic metabolism was significantly correlated with the harvest month which is in turn depended on growth rate. Thus, the high phenolic contents recorded in spring can be attributed to the development of young leaves, while the results for autumn indicate older, more mature leaves with a lower biosynthetic activity. This pattern may be explained by the fact that the *P. atlantica* leaves enhance their carbohydrate demands for regrowth and thus cause a tradeoff with secondary metabolite synthesis leading to a decrease in phenolic compounds. Our results are in agreement with Waterman and Mole (1994), who reported that in general young leaves have higher levels of mechanical and chemical defenses than mature.

Varying weather conditions also provide an explanation for the seasonal variation observed in the content of phenolic compounds. Biosynthesis of phenolic compounds is known to be sensitive to temperature and light, which reflects the possible role of these compounds in the photoprotection of plants (Yang et al. 2013). However, the lower temperatures in spring (Table 1) may also cause photo-oxidative stress, due to the limited activity of photo-synthetic enzymes, and therefore induce an increase in TPC levels (Close & McArthur 2002). This might be a reason for the higher concentration of TPC in April and May for Ain oussera and Laghouat regions, respectively. This observation is in agreement with another report that suggests that the level of phenylalanine ammonia lyase, an important enzyme involved in phenolic biosynthesis, increases considerably at lower temperatures (Karami et al. 2013).

In fact, the temperature is not the only factor that could interfere with the TPC level in leaves. Mechanisms that also induce seasonal variations may include one or both of the following environmental conditions: day length and visible light exposure, which vary according to the season (Table 1). In both regions, in the period June till October, when the leaves grow and the content of the phenolic compounds decreases, the day length and the duration of visible light exposure decrease. This might indicate that increasing day length and visible light exposure favor the biosynthesis of the TPC (June). However, in the considered regions the day length and visible light exposure differences are rather limited (about 3 h maximally).

Our data thus may suggest that high temperature and shorter day length with shorter visible light exposure can reduce the content of phenolic compounds in *P. atlantica* leaves and that the content of these compounds is lower in a season (autumn) associated with such growing conditions. The varying phenolic concentrations in *P. atlantica* leaves confirm the influence of both harvest season and climate factors on the production and release of these metabolites. In face of the obtained results, *P. atlantica* leaves used in traditional medicine should be collected during spring (April–May) in the considered regions in order to achieve maximal phenolic contents.

The total flavonoid and condensed tannins concentrations were relatively constant during the harvesting period (Tables 3 and 4). It was also observed that the contents of these compounds are rather similar between both regions. In contrast to the temperature, there were also few differences in visible light exposure and day length in both regions during the considered period.

Similar to results found in this study, Cohen et al. (2012) noted that there is no consistent relationship between temperature and CTC accumulation across three seasons. Flavonoid and condensed tannins biosynthesis is activated by plants as response to light energy and their accumulation has been demonstrated to serve, among other roles, as a protective shield to leaves, preventing molecular and cellular damage (Edreva 2005; Koyama et al. 2012). Most often kaempferol and quercetin are found glycosylated in *P. atlantica* leaves (Mosharrafa et al. 1999) which has been presented as a strategy for plant fitness against light energy (Harborne & Williams 2000). Our results mainly confirm the absence of a trend in FC and CTC concentrations in *P. atlantica* leaves even though significant differences may be related directly to photo-oxidative stress occurring at give moments in the growing season.

Moreover, when comparing our results with other published information (Gourine et al. 2010), it is worth noticing that both growing regions are located between 33 and 36° of the northern latitude. The declination of the sun is low, but the day length during the growth season is longer than in areas further south (Yang et al. 2013). This may be a cause of the selective metabolic differences in phenolic, flavonoid and condensed tannins contents in various published experiments.

Both the harvest month and the growing region manifest significant differences in the RSA of *P. atlantica* leaves. The leaves grown in the Ain oussera region have a significantly higher radical scavenging activity than those from the Laghouat region (Table 5). *Pistacia atlantica* leaves from the Ain oussera region were grown in arid climatic conditions, which likely describe the best environment for growth and development of *P. atlantica*, creating leaves with high-antiradical activity. The high free radical scavenging activity observed in the Ain oussera region is probably related to the lower temperature than in the Laghouat region (Table 1). This meteorological condition can enhance antiradical activity (Rodrigues et al. 2011). Our results mainly confirm the absence of a seasonal trend in scavenging antiradical activity in the *P. atlantica* leaves. This might indicate that the temperature is not the only factor that could interfere with the DPPH antioxidant activity results. Most of the responsible phytochemicals are produced in response to external stimuli, such as light intensity, moisture stress, day and night temperatures, rainfall, drought and their duration (Ncube et al. 2012). It is therefore possible that, depending on the harvest month, the content and presence of these bioactive compounds could vary, in parallel with the presence or absence of the stimuli, resulting in antiradical activity. The strongest DPPH radical scavenging potential was obtained from leaves of Ain oussera, harvested in June and September. Therefore, their extracts could be used as radical scavenging agents, acting as primary antioxidants.

In terms of reducing power capacity the variation was similar in both regions. The highest antioxidant capacity was recorded in spring (April–May). Then a continuous decrease was observed till autumn (September–October). The seasonal pattern in the reducing power capacity resembled to that observed for the total phenolic content (*r* = 0.337, see higher).

Hence, the male leaves collected from both regions appeared to have systematically stronger reducing power capacity than the female. Thus male leaves preferably are collected as crude medicine. However, for the DPPH results higher activities are seen in the Laghouat area, while for the reducing power activity rather the opposite is observed.

Hence it is likely that the morphological parameters of a leaf, such as epidermis, cuticle, palisade parenchyma, sizes, thickness and density, from a given region have played a key role in its antioxidant activity. It has been reported that the density of *P. atlantica* leaves collected from an arid site was higher than from a semi-arid site. In addition, increased aridity resulted in a reduction of leaf sizes, while their epidermis, cuticle, palisade parenchyma and total thickness increased (Said et al. 2011; Belhadj et al. 2011).

Differences in the antioxidant assay results could be due to the presence of compounds having a different affinities to react with DPPH or K_3_Fe(CN)_6_. Duh et al. (1999) reported that significant correlations between phenolics compounds content and antioxidant assays could support the hypothesis of the contribution of these compounds to the total antioxidant capacity of plant extracts. According to this hypothesis, it can be concluded that the phenolic compounds of *P. atlantica* could be main components responsible for the free radical scavenging activity and the reducing ability of these leaves. Furthermore, the condensed tannins may serve as a good source of antioxidant capacity as they have ferric reducing ability. The absence of correlation in this study can be assumed with *r*-values ranging from 0.048 to 0.250. However, our finding showed that the lack of correlation between FC and the reducing power, and between FC and DPPH may be linked to the assumption that most of the flavonoids in *P. atlantica* leaves were in their glycoside forms (Mosharrafa et al. 1999) and thus less effective compared to their aglycone forms (Anwar et al. 2013). Hence, the lack of correlation between CTC and DPPH is in agreement with Xu et al. (2012) and Chew et al. (2011), who reported that low molecular weight antioxidants were more effective as DPPH scavengers. Condensed tannins are high molecular weight polymers and, consequently, may be ineffective as DPPH scavengers. The absence of a significant correlation between RSA and RPC (see results) can be explained by the fact that both assays are based on two different mechanisms.

**Figure 6.**
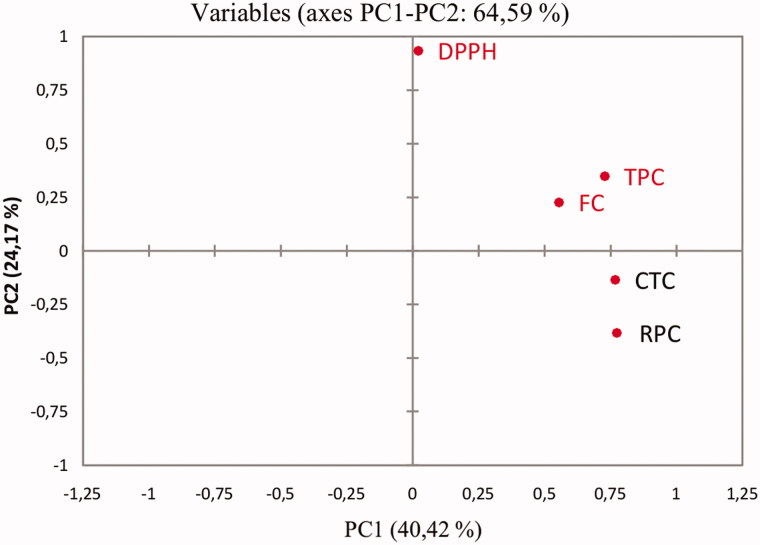
PC1-PC2 loading plot after autoscaling of the variables TPC, FC, CTC, DPPH and RPC.

Principal component analysis (PCA) was conducted to confirm any relationships among the analyzed variables and to visualize the multivariate data. PCA provides an exploratory data analysis and allows visualizing the data. PCA was applied to the data set of the 28 samples represented by the variables total phenolic, flavonoid, and condensed tannin contents, and their antioxidant activities. The first two principal components explained 64.6% of the total data variance. The PC1-PC2 loading plot is presented in Figure 6 and allows revealing information about the responses TPC, FC, CTC, DPPH, and RPC. The loading plot results confirm that the variable DPPH behaves differently from the rest; that RPC is more related to TPC and CTC, and that RPC and DPPH behave very differently.

**Figure 7.**
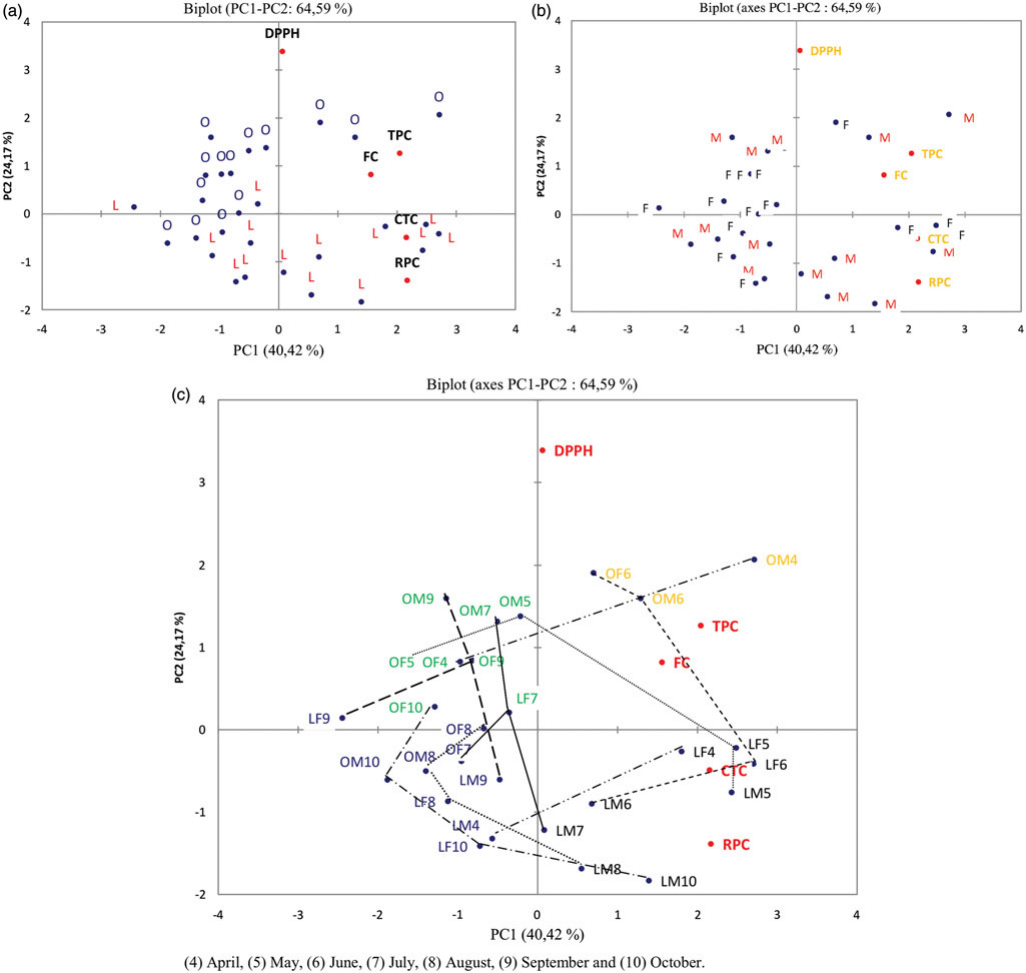
PC1-PC2 biplot, showing 28 *P. atlantica* samples labeled according to (a) harvest regions, (b) the genders, and (c) the harvest months.

PCA also allowed gaining an overview of the similarities and differences between regions genders and harvest months, when samples are labeled accordingly. The PC1-PC2 biplot is given in Figure 7 and provides information about both samples and variables (responses). The distance between two samples on a biplot is directly proportional to their degree of similarity or difference. Figure 7(a) shows that the samples from the Laghouat region (L) are mostly situated at the lower side of the score plot (low PC2 scores), while those from the Ain oussera region (O) are situated in the upper side of the plot (high PC2 scores). This difference is mainly caused by the variables, RPC and DPPH. For RPC, larger values are found for the samples from the Laghouat region (Table 6). On the other hand, DPPH values were higher for the samples of the Ain oussera region (Table 5). It could be concluded that the growing region differentiates the *P. atlantica* leaves, mainly on the basis of their antioxidant activities.

Figure 7(b) illustrates the PC1-PC2 biplot of the samples labeled according to gender. The two genders could not be differentiated on the plot. This result confirms the earlier finding that TPC, FC, CTC, RPC and RSA are non-significantly affected by the factor gender.

Having a closer look at Figure 7(c) indicates some seasonal tendency along PC1. Spring samples (months 4–6) tended to be situated at higher PC1 values, while autumn (months 9–10) samples mainly are situated at lower PC1 values. The results observed in the PC1–PC2 biplot (Figure 7(c)) thus differentiates to a certain degree *P. atlantica* leaves according to their harvest months.

## Conclusion

Our data revealed the changes in phytochemical contents along with the antioxidant activities of *P. atlantica* leaves as a function of harvest month, growing region and gender. The multivariate and statistical analyses of the results suggest that the phenolic compounds, flavonoid and condensed tannins contents, as well as the antioxidant activities were to a certain extent affected by the factor harvest month. The factor growing region also results in clearly different samples, where the difference is mainly due to the antioxidant activities. On the contrary, the factor gender did not seem to influence these responses. Furthermore, the correlation observed between the antioxidant assay results and the phenolic as well as the condensed tannins contents though limited are indications that these compounds are among the predominant sources of the antioxidant activity in *P. atlantica* leaves.

The present results may provide a basis for scientifically deciding on the optimal *P. atlantica* leaf-harvesting time and on the more optimal region for collecting.

## References

[CIT0001] Adhami VM, Muktar H. 2013 Human cancer chemoprevention hurdles and challenges, In: Pezzuto JM, Suh N, eds. Natural products in cancer prevention and therapy. Berlin: Springer; p. 204–217.

[CIT0002] Alov P, Tsakovska I, Pajeva I. 2015 Computational studies of free radical-scavenging properties of phenolic compounds. Curr Top Med Chem. 15:85–104. 25547098 10.2174/1568026615666141209143702 PMC4462847

[CIT0003] Anwar F, Shaheen N, Shabir G, Ashraf M, Alkharfy KM, Gilani AH. 2013 Variation in antioxidant activity and phenolic and flavonoid contents in the flowers and leaves of Ghaneri (*Lantana camara* L.) as affected by different extraction solvents. Int J Pharmacol. 9:442–453.

[CIT0004] Belhadj S, Derridj A, Moriana A, Gijon MDC, Mevy JP, Gauquelin T. 2011 Comparative analysis of stomatal characters in eight wild atlas pistachio populations (*Pistacia atlantica* Desf.; Anacardiaceae). Int Res J Plant Sci. 2:60–69.

[CIT0005] Belhadj S. 2002 Geographical distribution of *P. atlantica* Desf. Algeria Acta Hort. 591:499–503.

[CIT0006] Ben Ahmed Z, Yousfi M, Viaene J, Dejaegher B, Demeyer K, Mangelings D, Vander Heyden Y. 2016 Antioxidant activities of *Pistacia atlantica* extracts modeled as a function of chromatographic fingerprints in order to identify antioxidant markers. Microchem J. 128:208–217.

[CIT0007] Benhammou N, Bekkara FA, Panovska TK. 2008 Antioxidant and antimicrobial activities of the *Pistacia lentiscus* and *Pistacia atlantica* extracts. Afr J Pharm Pharmacol. 2:22–28.

[CIT0008] Boeing JS, Barizão ÉO, Costa de Silva B, Montanher PF, Almeida VDC, Visentainer JV. 2014 Evaluation of solvent effect on the extraction of phenolic compounds and antioxidant capacities from the berries: application of principal component analysis. Chem Cent J. 8:1–48. 25246942 10.1186/s13065-014-0048-1 PMC4158270

[CIT0009] Brunetti C, Guidi L, Sebastiani F, Tattini M. 2015 Isoprenoids and phenylpropanoids are key components of the antioxidant defense system of plants facing severe excess light stress. Environ Exp Bot. 119:54–62.

[CIT0010] Chew KK, Khoo MZ, Ng SY, Thoo YY, Wan Aida WM, Ho CW. 2011 Effect of ethanol concentration, extraction time and extraction temperature on the recovery of phenolic compounds and antioxidant capacity of *Orthosiphon stamineus* extracts. Int Food Res J. 18:571–578.

[CIT0011] Chupin L, Motillon C, Charrier-El Bouhtoury F, Pizzi A, Charrier B. 2013 Characterisation of maritime pine (*Pinus pinaster*) bark tannins extracted under different conditions by spectroscopic methods, FTIR and HPLC. Ind Crop Prod. 49:897–903.

[CIT0012] Close DC, McArthur C. 2002 Rethinking the role of many plant phenolics-protections from photodamage not herbivores? Oikos. 99:166–172.

[CIT0013] Cohen SD, Tarara JM, Gambetta GA, Matthews MA, Kennedy JA. 2012 Impact of diurnal temperature variation on grape berry development, proanthocyanidin accumulation, and the expression of flavonoid pathway genes. J Exp Bot. 63:2655–2665. 22268158 10.1093/jxb/err449 PMC3346226

[CIT0014] da Silva SM, Koehnlein EA, Bracht A, Castoldi R, de Morais GR, Baesso ML, Peralta RM. 2014 Inhibition of salivary and pancreatic α-amylases by a pinhão coat (*Araucaria angustifolia*) extract rich in condensed tannin. Food Res Intern. 56:1–8.

[CIT0015] de-Faria FM, Almeida ACA, Luiz-Ferreira A, Dunder RJ, Takayama C, da Silva M, Souza-Brito ARM. 2012 Mechanisms of action underlying the gastric antiulcer activity of the *Rhizophora mangle* L. J Ethnopharmacol. 139:234–243. 22100564 10.1016/j.jep.2011.11.007

[CIT0016] Du L, Shen Y, Zhang X, Prinyawiwatkul W, Xu Z. 2014 Antioxidant-rich phytochemicals in miracle berry (*Synsepalum dulcificum*) and antioxidant activity of its extracts. Food Chem. 153:279–284. 24491731 10.1016/j.foodchem.2013.12.072

[CIT0017] Duh PD, Tu YY, Yen GC. 1999 Antioxidant activity of water extract of Harng Jyur (*Chrysanthemum morifolium* Ramat). LWT-Food Sci Technol. 32:269–277.

[CIT0018] Edreva A. 2005 The importance of non-photosynthetic pigments and cinnamic acid derivatives in photoprotection. Agric Ecosyst Environ. 106:135–146.

[CIT0019] Fonseca JM, Rushing JW, Thomas RL, Riley MB, Rajapakse NC. 2007 Post-production stability of parthenolide in feverfew (*Tanacetum parthenium*). J Herbs Spices Med Plants. 12:139–152.

[CIT0020] El Zerey-Belaskri A, Benhassaini H. 2016 Morphological leaf variability in natural populations of *Pistacia atlantica* Desf. subsp. *atlantica* along climatic gradient: new features to update *Pistacia atlantica* subsp. *atlantica* key. Int J Biometeorol. 60:577–589. 26522787 10.1007/s00484-015-1052-4

[CIT0021] Gourine N, Yousfi M, Bombarda I, Nadjemi B, Stocker P, Gaydou EM. 2010 Antioxidant activities and chemical composition of essential oil of *Pistacia atlantica* from Algeria. Ind Crop Prod. 31:203–208.

[CIT0022] Harborne JB, Williams CA. 2000 Advances in flavonoid research since 1992. Phytochemistry. 55:481–504. 11130659 10.1016/s0031-9422(00)00235-1

[CIT0023] Kannan RRR, Arumugam R, Thangaradjou T, Anantharaman P. 2013 Phytochemical constituents, antioxidant properties and *p*-coumaric acid analysis in some seagrasses. Food Res Intern. 54:1229–1236.

[CIT0024] Karami Z, Mirzaei H, Emam-Djomeh Z, Sadeghi Mahoonak AR, Khomeiri M. 2013 Effect of harvest time on antioxidant activity of *Glycyrrhiza glabra* root extract and evaluation of its antibacterial activity. Int Food Res J. 20:2951–2957.

[CIT0025] Koyama K, Ikeda H, Poudel PR, Goto-Yamamoto N. 2012 Light quality affects flavonoid biosynthesis in young berries of Cabernet Sauvignon grape. Phytochemistry. 78:54–64. 22455871 10.1016/j.phytochem.2012.02.026

[CIT0026] Leong LP, Shui G. 2002 An investigation of antioxidant capacity of fruits in Singapore markets. Food Chem. 76:69–75.

[CIT0027] Li JE, Fan ST, Qiu ZH, Li C, Nie SP. 2015 Total flavonoids content, antioxidant and antimicrobial activities of extracts from *Mosla chinensis* Maxim. cv. Jiangxiangru. LWT-Food Sci Technol. 64:1022–1027.

[CIT0028] Liu S, Huang H. 2014 Assessments of antioxidant effect of black tea extract and its rationals by erythrocyte haemolysis assay, plasma oxidation assay and cellular antioxidant activity (CAA) assay. J Funct Foods. 18:1095–1105.

[CIT0029] Lou SN, Lin YS, Hsu YS, Chiu EM, Ho CT. 2014 Soluble and insoluble phenolic compounds and antioxidant activity of immature calamondin affected by solvents and heat treatment. Food Chem. 161:246–253. 24837947 10.1016/j.foodchem.2014.04.009

[CIT0030] Mosharrafa SAM, Kawashti SA, Saleh NAM. 1999 Flavonoids of *Pistacia atlantica* (Desf.). Bull Na Res Centre Egypt. 24:109–114.

[CIT0031] Ncube B, Finnie JF, Van Staden J. 2012 *In vitro* antimicrobial synergism within plant extract combinations from three South African medicinal bulbs. J Ethnopharmacol. l139:81–89. 10.1016/j.jep.2011.10.025 22075455

[CIT0032] Nieva‐Echevarría B, Manzanos MJ, Goicoechea E, Guillén MD. 2015 2, 6‐Di‐*tert*‐butyl‐hydroxytoluene and its metabolites in foods. Comp Rev Food Sci Food Safety. 14:67–80. 10.1111/1541-4337.12121 33401811

[CIT0033] Nyanhongo GS, Sygmund C, Ludwig R, Prasetyo EN, Guebitz GM. 2013 An antioxidant regenerating system for continuous quenching of free radicals in chronic wounds. Eur J Pharm Biopharm. 83:396–404. 23153671 10.1016/j.ejpb.2012.10.013

[CIT0034] Rodrigues AS, Pérez-Gregorio MR, García-Falcón MS, Simal-Gándara J, Almeida DPF. 2011 Effect of meteorological conditions on antioxidant flavonoids in Portuguese cultivars of white and red onions. Food Chem. 124:303–308.

[CIT0035] Said SA, Fernandez C, Greff S, Derridj A, Gauquelin T, Mevy JP. 2011 Inter-population variability of leaf morpho-anatomical and terpenoid patterns of *Pistacia atlantica* Desf. ssp. *atlantica* growing along an aridity gradient in Algeria. Flora. 206:397–405.

[CIT0036] Stanković MS, Petrović M, Godjevac D, Stevanović ZD. 2015 Screening inland halophytes from the central balkan for their antioxidant activity in relation to total phenolic compounds and flavonoids: Are there any prospective medicinal plants? Arid Environ. 120:26–32.

[CIT0037] Suriano S, Alba V, Tarricone L, Di Gennaro D. 2015 Maceration with stems contact fermentation: Effect on proanthocyanidins compounds and color in Primitivo red wines. Food Chem. 177:382–389. 25660901 10.1016/j.foodchem.2015.01.063

[CIT0038] Vagiri M, Conner S, Stewart D, Andersson SC, Verrall S, Johansson E, Rumpunen K. 2015 Phenolic compounds in blackcurrant (*Ribes nigrum* L.) leaves relative to leaf position and harvest date. Food Chem. 172:135–142. 25442534 10.1016/j.foodchem.2014.09.041

[CIT0039] Vongsak B, Sithisarn P, Mangmool S, Thongpraditchote S, Wongkrajang Y, Gritsanapan W. 2013 Maximizing total phenolics, total flavonoids contents and antioxidant activity of *Moringa oleifera* leaf extract by the appropriate extraction method. Ind Crop Prod. 44:566–571.

[CIT0040] Waterman PG, Mole S. 1994 Analysis of phenolic plant metabolites. Oxford (UK): Blackwell Scientific, Publications; p. 85–87.

[CIT0041] Weather Underground Weather underground provides c2010–2016. San Francisco, California: The Weather Company, LLC. Available from: http://french.wunderground.com/history/

[CIT0042] Xu Y, Sismour EN, Parry J, Hanna MA, Li H. 2012 Nutritional composition and antioxidant activity in hazelnut shells from US‐grown cultivars. Int J Food Sci Technol. 47:940–946.

[CIT0043] Yang B, Zheng J, Laaksonen O, Tahvonen R, Kallio H. 2013 Effects of latitude and weather conditions on phenolic compounds in currant (*Ribes* spp.) cultivars. J Agric Food Chem. 61:3517–3532. 23480522 10.1021/jf4000456

[CIT0044] Yousfi M, Djeridane A, Nadjemi B, Boutassouna D, Stocker P, Vidal N. 2006 Antioxidant activity of some Algerian medicinal plants extracts containing phenolic compounds. Food Chem. 97:654–660.

[CIT0045] Zarshenas MM, Arabzadeh A, Tafti MA, Kordafshari G, Zargaran A, Mohagheghzadeh A. 2013 Application of herbal exudates in traditional Persian medicine. Galen J Med. 1:78–83.

